# Higher scores on autonomic symptom scales in pediatric patients with neurodevelopmental disorders of known genetic etiology

**DOI:** 10.1002/brb3.2813

**Published:** 2022-11-24

**Authors:** Antoinette S DiCriscio, KE Wain, J Smith, D Beiler, LK Walsh, K Holdren, Vanessa Troiani

**Affiliations:** ^1^ Geisinger Health System Autism & Developmental Medicine Institute (ADMI) Lewisburg Pennsylvania USA; ^2^ Department of Imaging Science and Innovation Center for Health Research, Geisinger Danville Pennsylvania USA; ^3^ Neuroscience Institute, Geisinger Danville Pennsylvania USA; ^4^ Department of Basic Sciences Geisinger Commonwealth School of Medicine Scranton Pennsylvania USA

**Keywords:** autism spectrum disorder, autonomic nervous system, developmental brain dysfunction, individual differences

## Abstract

**Introduction:**

Features of underlying autonomic dysfunction, including sleep disturbances, gastrointestinal problems, and atypical heart rate, have been reported in neurodevelopmental conditions, including autism spectrum disorder (ASD). The current cross‐sectional, between‐groups study aimed to quantify symptoms of autonomic dysfunction in a neurodevelopmental pediatric cohort characterized by clinical diagnoses as well as genetic etiology.

**Method:**

The Pediatric Autonomic Symptom Scales (PASS) questionnaire was used to assess autonomic features across a group of patients with clinical neurodevelopmental diagnoses (NPD; *N* = 90) and genetic etiologies. Patients were subdivided based on either having a clinical ASD diagnosis (NPD‐ASD; *n* = 37) or other non‐ASD neurodevelopmental diagnoses, such as intellectual disability without ASD, speech and language disorders, and/or attention deficit hyperactivity disorder (NPD‐OTHER; *n* = 53). Analyses focused on characterizing differences between the NPD group compared to previously published reference samples, as well as differences between the two NPD subgroups (NPD‐ASD and NPD‐OTHER).

**Results:**

Our results indicate higher PASS scores in our NPD cohort relative to children with and without ASD from a previously published cohort. However, we did not identify significant group differences between our NPD‐ASD and NPD‐OTHER subgroups. Furthermore, we find a significant relationship between quantitative ASD traits and symptoms of autonomic function.

**Conclusion:**

This work demonstrates the utility of capturing quantitative estimates of autonomic trait dimensions that may be significantly linked with psychosocial impairments and other core clinical features of ASD.

## Graphical abstract

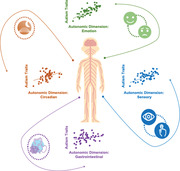

## 1 BACKGROUND

Although not a part of the core diagnost criteria, symptoms related to underlying dysfunction of the autonomic nervous system (ANS) are commonly observed in patients with autism spectrum disorder (ASD), including sleep disturbances, gastrointestinal problems, and atypical heart rate (Aldinger et al., 2015; Devnani & Hegde, 2015; Kotagal & Broomall, 2012; Kushki et al., 2013; Liu et al., 2006; Mayes & Calhoun, 2009; Neuhaus et al., 2014; Patriquin et al., 2013). The ANS (i.e., the peripheral nervous system) maintains homeostasis in the body via a synchronous balance of sympathetic and parasympathetic activity across functions including heart rate, digestion, respiration, sleep, and sensory processing. Failure of one of the parasympathetic or sympathetic components of the ANS can result in malfunction or disruption of the system. Recent work has described a dysregulated ANS as a potential biological driver of anxiety, sensory disintegration, and psychosocial impairments that are significant clinical features of atypical neurodevelopment, including ASD (Appelhans & Luecken, 2006; Goodwin et al., 2006; Kushki et al., 2013; Kushki et al., 2014; Patriquin et al., 2013; Patriquin et al., 2019; Quintana et al., 2012).

Over the past 15 years, a number of studies have highlighted the role of the ANS in the underlying pathophysiology of brain‐related disorders and assessed autonomic dysregulation and atypical circadian function associated with neurodegenerative disease states (Germain & Kupfer, 2008; Musiek et al., 2015), various forms of psychopathology (Sgoifo et al., 2015; Videnovic & Zee, 2015; Wirz‐Justice et al., 2009; Wulff et al., 2012), and neurodevelopmental conditions such as ASD (Anderson & Colombo, 2009; Bellato et al., 2020; Bharath et al., 2019; Dell'Osso et al., 2022; Rukmani et al., 2016). Studies have reported atypical autonomic responses, including heart rate variability, skin conductance, pupillary response, and neuroendocrine markers (Gabriels et al., 2013; Kushki et al., 2013; Ming et al., 2005; Poquérusse et al., 2018; Song et al., 2016; Tordjman et al., 2014; Wang et al., 2016). These differences in physiological outputs have been interpreted as peripheral biological indicators of discordant autonomic arousal. The observed differences in measures of cardiac output, electrodermal activity, and pupil reactivity have also been theoretically and quantitatively linked to deficits in social cognition and social impairments (Appelhans & Luecken, 2006; Cheshire, 2012; Quintana et al., 2012). More specifically, task‐induced measures of heart rate and sinus arrhythmia have been associated with impaired social reciprocity (Neuhaus et al., 2014; Patriquin et al., 2013), as well as social and emotional perception, cognitive abilities, and restrictive and repetitive behaviors (Billeci et al., 2018; Condy et al., 2017; Kushki et al., 2014; Soker‐Elimaliah et al., 2020). From this lens, differences in physiological outputs associated with ANS dysfunction have been attributed to underlying neurobiological differences and core clinical features of ASD (Dinalankara et al., 2017; Kushki et al., 2014; Ming et al., 2005; Pace et al., 2016; Patriquin et al., 2013). Taken together, this research highlights autonomic function as a possible indicator of dysregulated neurological states that can be linked with atypical neurodevelopment and ASD trait dimensions.

It is standard of care to offer diagnostic genetic testing to patients with ASD and/or other neurodevelopmental disorders to assess for genetic etiologies. Known genetic causes of ASD and other neurodevelopmental disorders are etiologically heterogeneous and include copy number variants (CNVs), sequence‐level variants in single genes (SNVs), and epigenetic alterations (for review, see Rylaarsdam and Guemez Gamboa (2019)). Importantly, many genetic disorders confer high risk for a broad spectrum of neurodevelopmental/psychiatric disorders (NPD), which represent variable expressivity of underlying developmental brain dysfunction (Moreno‐De‐Luca et al., 2013). For example, recurrent pathogenic CNVs (e.g., 22q11.2, 16p11.2) are associated with several psychiatric and neurodevelopmental disorders (Gudmundsson et al., 2019; Martin et al., 2020; Stefansson et al., 2014), including ASD (Hanson et al., 2015; Kates et al., 2007; Niklasson et al., 2009; Shinawi et al., 2010). When examining common variation across the genome, several studies have also identified shared genetic risk across multiple psychiatric and neurodevelopmental disorders, highlighting the role of shared genetic risk factors for clinical diagnoses and phenotypic variability across NPD (Cross‐Disorder Group of the Psychiatric Genomics Consortium, 2013; Lee et al., 2019). Thus, there is increased recognition that genetic influences on psychiatric and neurodevelopmental disorders are pleiotropic and transcend diagnostic boundaries.

Individuals with NPD‐related genetic disorders have been reported to have symptoms such as atypical sleep–wake cycles or disrupted patterns of sleep, gastrointestinal and digestive disturbances, and others that can be interpreted as signs of ANS dysfunction (Brunetti‐Pierri et al., 2008; Cerminara et al., 2021; Kamara et al., 2021; Leader et al., 2021; Shayota & Elsea, 2019). However, there is little known regarding the connection between ANS‐related symptoms and genetic variation associated with atypical neurodevelopment, including ASD (Dell'Osso et al., 2022; Hu et al., 2009; Lorsung et al., 2021). Thus, a more comprehensive understanding of the presence of a common transdiagnostic domain, such as ANS‐related symptoms, in children with neurodevelopmental disorders may lead us closer to the underlying biology that drives behavioral differences in heterogeneous disorders. This transdiagnostic approach aligns with recent shifts in clinical outcomes and behavioral research to focus more on quantitative symptom scales or trait dimensions in a cross‐disorder manner, outside of traditional clinical taxonomy (Dalgleish et al., 2020; Gentes & Ruscio, 2011; Mahoney & McEvoy, 2012). Although it is ideal to capture symptoms via direct measurement, one can also capture variance in a variety of symptom domains using validated self‐ or parental‐report questionnaires. Quantitative measures of autonomic features have been previously utilized in patients with other neurological disorders (Adler et al., 2018; Aziz et al., 2010; Damian et al., 2012), although a majority of previous studies are based on adults (Damian et al., 2012; Sletten et al., 2012) or focused on one symptom domain (Morlino et al., 2019). Studies of ANS function within neurodevelopmental populations have not typically used ANS‐specific surveys, but rather relied on measures of anxiety, sensory processing, or sleep–wake cycles to assess features of arousal that were interpreted to reflect dysregulated ANS function (Keith et al., 2019; Lawson et al., 2020; Wiggs & Stores, 1996; Wiggs & Stores, 2004). One study used an ANS‐specific scale, the Composite Autonomic Symptoms Scales (COMPASS‐31), and noted increased symptoms of autonomic dysfunction in adolescents and young adults with ASD and a relationship between COMPASS‐31 scores and ASD traits (Lawson et al., 2020; Sletten et al., 2012). Another previous study used the Pediatric Autonomic Symptom Scales (PASS; adapted from the CASS and COMPASS‐31) (Sletten et al., 2012; Suarez et al., 1999) to demonstrate that features of autonomic dysfunction are elevated in a small cohort of children with ASD (Ming et al., 2011) (*n* = 18), for which genetic etiology was unknown.

Here, we capture autonomic symptoms as measured by the PASS (Ming et al., 2011) in a clinically characterized, pediatric cohort with genetic NPD etiologies, a subset of whom have a clinical ASD diagnosis. Although our group analyses focus on clinical diagnoses of ASD versus other non‐ASD NPD, we include comprehensive information on genetic etiology in this genotyped cohort in order to promote future research on such highly heterogeneous, real‐world samples. The overall objective of the current investigation was to quantify symptoms of autonomic dysfunction in a neurodevelopmental pediatric cohort characterized based on both clinical diagnoses as well as clinical genetic etiology. Based on previous studies of autonomic features in neurodevelopmental populations, we hypothesized that individuals with a genetic NPD etiology would demonstrate an increase in atypical symptoms of ANS function as compared to a published reference sample of children with and without ASD (Ming et al., 2011). Additionally, we aimed to assess individual differences in ANS function that may scale with the presence and severity of ASD symptoms across children with and without a clinical diagnosis of ASD and/or other NPDs. Research has demonstrated the link between autonomic dysregulation and psychosocial features, including core diagnostic traits of ASD (Appelhans & Luecken, 2006; Goodwin et al., 2006; Kushki et al., 2013; Kushki et al., 2014; Patriquin et al., 2013; Patriquin et al., 2019; Quintana et al., 2012). As a part of the current study, we characterized the direct relationship between features of autonomic dysfunction and symptom severity across ASD trait dimensions.

## 2 METHODS

### 2.1 Participants

The NPD cohort in the current cross‐sectional, between‐groups study was identified based on documentation of a diagnosis of a targeted NPD‐related genetic syndrome and current enrollment in an ongoing research protocol at the authors’ home institution. All eligible participants had consented to an ongoing research protocol, approved by the authors’ home institution's institutional review board (IRB) and ethics committee, that allowed for recontact and participation in an online phenotyping battery, including parent‐report measures (described below). Data were collected between April 2018 and November 2019. Individual genetic syndromes are reported in Table 1, with the most common syndromes including 16p11.2 (BP4‐BP5) deletion (del), *n* = 14; 17p11.2 del, *n* = 8; 17q12 del, *n* = 9; and Trisomy 21, *n* = 11. Patients at Geisinger's Autism & Developmental Medicine Institute (ADMI) undergo assessment by a multi‐disciplinary team including neurodevelopment pediatricians, clinical psychologists, behavioral specialists, and speech pathologists. Assessment tools and/or structured interviews, such as the ADOS or ADI‐R, may be used but diagnoses are ultimately made by the clinicians using DSM‐5 criteria for ASD after a comprehensive evaluation of the patient. ADOS and ADI‐R scores were not available from the electronic health record for enough patients in the current sample and thus were not used in any analyses.

**TABLE 1:** Demographic information and a summary of genetic syndromes (copy number state, coordinates, and pathogenicity) included in the reported NPD sample (*N* = 90)

Chromosome region	*n*, Sex (M/F)	*n* = ASD	Copy number state	Accepted general coordinates (hg19/GRCh37)	ClinGen pathogenicity	$x\bar{\left(\sigma \right)}$	Min	Max
**Copy number losses**								
1q21.1 (BP3‐BP4, wo TAR region)	*n* = 4, 3 M	1	Deletion	chr1:146,577,486‐147,394,506 (hg19)	3	10.25 (6.65)	2	18
7q11.23	*n* = 1, F		Deletion	chr7:72,744,455‐74,142,510 (hg19)	3	3		
15q11.2 (BP1‐BP2)	n = 1, M	1	Deletion	chr15:22,832,519‐23,090‐897 (hg19)	2	13		
15q11.2q13.1	*n* = 1, M		Deletion	chr15:23,747,996‐28,379,874 (hg19)	3	18		
15q13.3 (BP3‐BP5)	*n* = 1, F		Deletion	chr15:29,156,959‐32,445,405 (hg19)	3	16		
16p11.2 (BP4‐BP5)	*n* = 14, 9 M	6	Deletion	chr16:29,649,997‐30,199,852 (hg19)	3	11.21 (4.14)	6	17
16p11.2 (*SH2B1*)	*n* = 2, 1 M	1	Deletion	chr16:28,822,635‐29,046,499 (hg19)	3	9.50 (4.95)	6	13
16p12.2 (*EEF2K*)	*n* = 1, F	1	Deletion	chr16:21,948,445‐22,430,804 (hg19)	2	11		
16p13.11	*n* = 1, F		Deletion	chr16:15,511,711‐16,292,265 (hg19)	3	12		
17p11.2	*n* = 8, 5 M	4	Deletion	chr17:16,810,028‐20,213,202	3	10.75 (4.56)	5	18
17q12	*n* = 9, 7 M	2	Deletion	chr17:34,815,072‐36,192,489 (hg19)	3	9.44 (5.00)	3	18
17q21.31	*n* = 1, F		Deletion	chr17:43,705,166‐44,164,880 (hg19)	3	4		
22q11.2 (3 Mb)	*n* = 3, 2 M		Deletion	chr22:18,912,231‐21,465,672 (hg19)	3	11.33 (5.51)	6	17
**Copy number gains**								
1q21.1 (BP2‐BP3, TAR region only)	*n* = 2, 1 M	1	Duplication	chr1:145,386,507‐145,748,064 (hg19)	1	8.50 (0.71)	8	9
1q21.1 (BP3‐BP4, wo TAR region)	*n* = 3, 2 M	1	Duplication	chr1:146,577,486‐147,394,506 (hg19)	3	8.00 (5.00)	3	13
1q21.1 (coordinates not available)	*n* = 1, M	1	Duplication	n/a	n/a	13		
15q11.1q13.3 (supernumerary isodicentric)	*n* = 2, 2 F		Triplication	variable	Not evaluated	8.00 (0.00)	8	8
15q11.2q13.1	*n* = 5, 2 M	3	Duplication	chr15:23,747,996‐28,379,874 (hg19)	3	14.20 (4.38)	7	17
15q11.2q13.1 (interstitial tandem triplication)	*n* = 1, F	1	Triplication	variable	Not evaluated	3		
15q11.2q13.3 (supernumerary isodicentric)	n = 1, F	1	Duplication	variable	Not evaluated	12		
15q13.3 (BP3‐BP5)	*n* = 1, M	1	Duplication	chr15:29,156,959‐32,445,405 (hg19)	1	11		
16p11.2 (*SH2B1*)	*n* = 1, M	1	Duplication	chr16:28,822,635‐29,046,499 (hg19)	1	3		
16p13.11	*n* = 3, 2 M	2	Duplication	chr16:15,511,711‐16,292,265 (hg19)	2	2.33 (0.58)	2	3
17q12	*n* = 7, 6 M	4	Duplication	chr17:34,815,072‐36,192,489 (hg19)	3	6.71 (3.09)	2	10
Trisomy 21,	*n* = 11, 7 M	2	Trisomy	chr21:1‐48,129,895 (hg19)	Not evaluated	8.91 (3.08)	4	13
22q11.2 (3 Mb)	*n* = 2, 2 M	1	Duplication	chr22:18,912,231‐21,465,672 (hg19)	3	10.00 (5.66)	6	17
**Intragenic sequence variant**								
17p11.2 (*RAI1* sequence variant)	*n* = 2, 2 F		Sequence variant	n/a	n/a	9.00(2.83)	7	11
**Other copy number variant**								
15q11.2q13.3	*n* = 1, M		Not available	variable	Not evaluated	7		

Average age ($x\bar{\left(\sigma \right)}$) as well as range of age for genetic syndrome is outlined in columns to the right.

All patients with qualifying clinical diagnoses are offered comprehensive clinical genetic testing and result follow‐up with the ADMI genetic counseling team. All clinical diagnoses, including ASD and any comorbidities, and genetic test results are entered into the patient's digital health record. The majority of patients (>85%) also consent/assent to a clinic‐wide research protocol, which allows researchers to access the patient's health record and recontact for additional research. Patients were eligible if they had received a clinical NPD diagnosis with a known genetic etiology and were recontacted to request completion of the parental report measures described below; clinical diagnoses and other parent‐report measures available in individuals’ EHR were also curated for this study. A total of 134 eligible participants completed and returned the PASS. However, due to factors including incomplete responses, age of the proband outside of the targeted age range, and missing clinical, diagnostic, and/or genetic information, data from *n* = 44 were excluded from analysis, resulting in our reported cohort of *N* = 90 NPD included in the current study. *N* = 90 (entire sample) were subdivided based on either having a clinical diagnosis of ASD (NPD‐ASD; *n* = 37) or another NPD diagnosis (NPD‐OTHER; *n* = 53). Other non‐ASD NPD diagnoses, present across NPD‐ASD *and* NPD‐OTHER subgroups, included attention deficit hyperactivity disorder (ADHD), anxiety, speech and language disorder, and mild‐to‐moderate intellectual disability (ID). All of the individuals within the NPD‐ASD subgroup had a diagnosis of ASD and at least one co‐occurring clinical diagnosis of another form of NPD (i.e., intellectual disability, ADHD, anxiety, etc.). To confirm, none of the individuals in the NPD‐OTHER had a clinical diagnosis of ASD; thus, this group comprised *only* individuals with other, non‐ASD forms on NPD (i.e., not ASD). Table S1 outlines the clinical diagnoses within each of the NPD‐ASD and NPD‐OTHER subgroups.

### 2.2 Parent‐report measures

All parent‐report measures outlined below were administered via an online phenotyping battery administered and maintained using REDCap. Parents and/or caregivers of consented patients received a personalized link, directing them to complete a comprehensive battery of various symptom questionnaires related to neurodevelopmental traits.

#### 2.2.1 Pediatric autonomic symptom scales (PASS)

**FIGURE 1:**
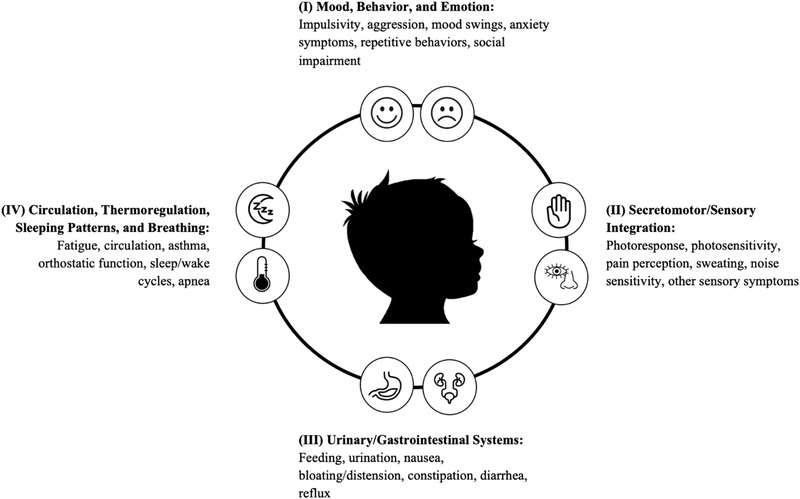
The Pediatric Autonomic Symptoms Scale (PASS). The PASS is an 80‐item parent‐report questionnaire of autonomic symptoms in children. Items are scored based on the presence (Yes = 1) or absence (No = 0) of symptoms. Items are summed resulting in a total score as well as four subscale scores: (I) Mood, Behavior, and Emotion; (II) Secretomotor/Sensory Integration; (III) Urinary/Gastrointestinal Systems; and (IV) Circulation, Thermoregulation, Sleep Patterns, and Breathing.

The PASS (Ming et al., 2011; Suarez et al., 1999) is a parent/caregiver report questionnaire designed to assess the severity of autonomic dysfunction across multiple systems in children. The questionnaire was adapted for use in pediatric cohorts based on the adult clinical research scale, the Composite Autonomic Symptom Scale (CASS) (Suarez et al., 1999). It includes 80 close‐ended questions from four section subscales grouped by the affected organ or organ systems—(I): Mood, Behavior, and Emotion; (II): Secretomotor/Sensory Integration; (III): Urinary/Gastrointestinal Systems; and (IV): Circulation, Thermoregulation, Sleeping Patterns, and Breathing (see Figure 1). The first type of question is based on the presence or absence of a symptom, such as “Have you noticed that your child seems to have difficulty seeing after coming out of a dark room?” If the answer represented dysfunction, it was scored as “1” whereas absence of the symptom or appropriate function was scored “0.” The second type of question assessed severity or frequency of the symptom, such as ‘‘Does your child urinate frequently, such as more than 10 times daily?’’ or ‘‘Does your child typically skip having a bowel movement for 2 days or more?’’ Item responses are summed, resulting in a Total Autonomic score and four subscale scores for each of the sections outlined above.

#### 2.2.2 The social responsiveness scale—2nd edition (SRS‐2)

The SRS‐2 is a 65‐item rating scale widely used to identify social impairment and persons at risk for ASD from 2.5 years to adulthood (Constantino & Frazier, 2013; Constantino & Gruber, 2005; Constantino & Gruber, 2012). The SRS offers the advantage of being sensitive to subclinical ASD behaviors in the general population. Each item, such as “avoids eye contact, or has unusual eye contact” or “would rather be alone than with others” is measured on a 4‐point (0–3) scale: 0 = not true, 1 = sometimes true, 2 = often true, and 3 = almost always true. Group average SRS Total t‐scores, a summary of participants with t‐scores across the mild, moderate, and severe ranges, and total raw and subscale scores with participant demographics are reported in Table 2. We used SRS raw scores in the current study as a proxy of ASD clinical features and to maximize phenotypic variability across our sample.

**TABLE 2:** Demographics and SRS scores in *N* = 90 NPD (55 males) as well as within specific subgroups established from Total NPD sample

				*Subgroups* defined from Total sample					
	*Total* NPD sample *N* = 90			NPD‐ASD *n* = 37 of 90			NPD‐OTHER *n* = 53 of 90		
	$\bar{x}\left(\sigma \right)$	Min	Max	$\bar{x}\left(\sigma \right)$	Min	Max	$\bar{x}\left(\sigma \right)$	Min	Max
**Age (in years)**	9.71 (4.50)	2	18						
**SRS Total *t*‐score**	74.51 (13.93)	40	105	82.52 (11.48)	53	89	67.55 (12.21)	50	72
*n* = *Mild range (T‐score 60 −65)*	*n* = 6			*n* = 1			*n* = 5		
*n* = *Moderate (T‐score 66 −75)*	*n* = 16			*n* = 5			*n* = 11		
*n* = *Severe (T‐score ≥ 76)*	*n* = 23			*n* = 16			*n* = 7		
**SRS Raw scores (*n* = 52; Male:33)**									
Total	93.65 (33.53)	11	161	114.39 (26.86)	50	73	77.21 (29.12)	50	64
SCI	75.90 (26.29)	9	128	90.96 (21.50)	50	85	63.97 (23.71)	50	82
RBRI	17.75 (8.32)	1	33	23.43 (6.67)	50	78	13.24 (6.61)	50	74
Social awareness	12.39 (4.11)	3	22	14.74 (3.26)	59	105	10.52 (3.77)	40	89
Social cognition	18.98 (6.71)	3	31	22.04 (4.47)	55	88	22.04 (4.47)	31	77
Social communication	31.14 (12.18)	0	58	37.48 (10.35)	57	83	26.10 (11.25)	48	77
Social motivation	13.40 (6.11)	2	27	16.69 (6.36)	50	79	10.79 (4.49)	50	74

In addition to clinical diagnoses and parent‐report measures, we curated outcomes of an additional available measure of co‐occurring clinical and behavioral features assessed via the Child Behavior Checklist (CBCL) from the EHR. See Table S2 for additional summary of demographic variables, including parent‐report measures and CBCL scores (school‐age and pre‐school ages). Although the CBCL scores were not part of our main hypotheses, we include them in Supporting Information to provide a more complete characterization of the range of this cohort's emotional and behavioral problems.

### 2.3 Analysis and statistical methods

Prior to carrying out formal analyses that addressed our primary research questions (outlined below), we assessed the distribution of our measures using a Shapiro–Wilk test of normality. PASS Total and subscale scores deviated from a normal distribution (*p*’s < .019, NS) with the exception of PASS (I) Mood, Behavior, and Emotion (*p* > .061, NS). Due to these results, non‐parametric tests were used to assess group comparisons. An alpha‐level of *p* < .05 was used across all analysis to identify significant results with appropriate correction for multiple comparisons for each stage of our analyses. We also assessed the inter‐correlations among PASS subscales in our reported cohort and noted significant relationships across all PASS subscales. Complete results from this analysis are reported in Table S3.

We analyzed participant data by grouping participants in several ways: (1) For cross‐sectional, between‐groups comparisons, we compared scores from the entire sample to a published reference sample of children with and without ASD from Ming et al. (2011) via Wilcoxon, exact sign procedures in R Studio with continuity correction for comparison between a continuous and discrete population variables. Since the reference sample included children in a 2–5 year age range (Ming et al., 2011) and the current sample included ages 2–18 years, we first confirmed that scores did not differ by age (see complete results from this analysis in Table S4). (2) In additional between‐groups comparisons, we also split the NPD group into two subgroups, those that had a clinical diagnosis of ASD (NPD‐ASD; *n* = 37) and those that had other, non‐ASD forms of NPDs (NPD‐OTHER; *n* = 53). Again, non‐parametric sign procedures were used for subgroup comparisons with appropriate corrections for multiple comparisons (i.e., Bonferroni methods were used for comparisons between NPD‐ASD and NPD‐OTHER; continuity correction was used for comparisons between the current cohort and previously published samples). Finally (3), across our larger *N* = 90 NPD cohort, we dimensionally assessed the relationship between individual differences in symptoms scales of ANS dysfunction and core clinical features of ASD using the SRS via partial correlation procedures controlling for chronological age and sex with correction for multiple comparisons via Bonferroni methods.

## 3 RESULTS

### 3.1 Differences in PASS scores in pediatric probands, children with ASD, and healthy controls

We first compared PASS scores collected in the *N* = 90 NPD group to a published reference sample of children with and without ASD (from Ming et al. (2011)). Children in the NPD group had higher PASS Total scores as compared to children with ASD (*p* = .003) and healthy controls (*p* < .0001) from the reference sample. This finding of higher PASS Total scores in the NPD group was also confirmed across all subscales (*p*’s < .041). See Table 3 for group average PASS scores and results of between group comparisons.

**TABLE 3:** Pediatric Autonomic Symptom Scale (PASS) scores on *N* = 90 NPD identified based on having a genetic syndrome that confers increased risk for ASD or other neurodevelopmental diagnosis

	*N* = 90 NPD			Ming et al., 2011		
				*n* = 18 ASD	*n* = 24 HC	
	$\bar{x}\left(\sigma \right)$7	Min	Max	$\bar{x}\left(\sigma \right)$	$\bar{x}\left(\sigma \right)$	*p*‐value
**Age**	9.71 (4.50)	2	18			
**PASS**						
**PASS Total**	24.51 (11.14)	3	68	20.6 (2.7)	6.0 (0.8)	^a^ *p* = .003* ^b^ *p* < .0001**
**Section I** ***Mood, Behavior, and Emotion (MBE)***	9.26 (4.50)	0	18	10.7 (0.8)	2.6 (0.3)	^a^ *p* = .004* ^b^ *p* < .0001**
**Section II** ***Secretomotor and Sensory Integration (SS)***	6.37 (3.78)	0	19	4.5 (0.8)	1.5 (0.3)	^a^ *p* < .0001** ^b^ *p* < .0001**
**Section III** ***Urinary and Gastrointestinal Systems (UG)***	4.81 (3.37)	0	15	3.8 (0.7)	1.3 (0.2)	^a^ *p* = .019* ^b^ *p* < .0001**
**Section IV** ***Circulation, Thermoregulation, and Sleep (CTS)***	4.08 (3.00)	0	17	3.1 (0.7)	0.9 (0.2)	^a^ *p* = .041* ^b^ *p* < .0001**

All *p*‐values reported below are based on results from Wilcoxon signed test with continuity correction.

^a^ Group comparisons between mean PASS scores in NPD in the current research as compared to mean PASS scores for the ASD group reported in Ming et al. (2011).

^b^ Group comparisons between mean PASS scores in NPD in the current research as compared to healthy controls reported in Ming et al. (2011).

* *p* < 0.05;.

** *p* < 0.001.

We also explored differences between subgroups of our cohort: those with a clinical diagnosis of ASD (NPD‐ASD, *n* = 37 of 90) versus those with non‐ASD NPD diagnoses (NPD‐OTHER, *n* = 53 of 90) (Table 4). NPD‐ASD had higher PASS Total scores as compared to NPD‐OTHER (*p* = .009) and higher scores relative to the published ASD reference sample (*p* = .0006). NPD‐ASD demonstrated significantly higher scores on the PASS (I) Mood, Behavior, and Emotion and (II) Secretomotor and Sensory Integration subscales as compared to NPD‐OTHER (*p*’s < .021), but not the other two PASS subscales (*p*’s > .516, NS). When compared to the Ming et al. (2011) ASD reference sample, NPD‐ASD also demonstrated significantly higher scores on the PASS (III) Secretomotor and Sensory Integration and (IV) Thermoregulation, Circulation, and Sleep subscales (*p*’s < .049), but not other subscales (*p* > .061, NS). Finally, no significant differences in PASS scores were found between NPD‐OTHER as compared to the Ming et al. (2011) ASD reference sample (*p*’s > .0625, NS), with the exception of the (I) Mood, Behavior, and Emotion subscale (*p* < .0001). Thus, symptoms of autonomic dysfunction, particularly in the (I) Mood, Behavior, and Emotion and (II) Secretomotor/Sensory Integration domains, are increased in children with a NPD‐related genetic etiology and an ASD diagnosis relative to those without ASD and compared to previously published samples of children with ASD of unknown etiology.

**TABLE 4:** PASS scores in NPD probands with ASD (*n* = 37 NPD‐ASD), probands with other forms of NPD (*n* = 53 NPD‐OTHER), and ASD subsample reported in Ming et al. (2011)

	*n* = 37 of 90 NPD‐ASD			*n* = 53 of 90 NPD‐OTHER			Ming et al. *n* = 18 ASD	
	$\bar{x}\left(\sigma \right)$	Min	Max	$\bar{x}\left(\sigma \right)$	Min	Max	$\bar{x}\left(\sigma \right)$	*p*‐value
**Age**	9.78 (4.91)	2	18	9.51 (4.29)	2	18		
**PASS**								
**PASS Total**	28.16 (11.75)	7	68	21.96 (10.02)	3	45	20.6 (2.7)	^a^ *p* = .0109* ^b^ *p* = .0006** ^c^ *p* = .5353, NS
**Section I** ***Mood, Behavior, and Emotion (MBE)***	11.32 (4.31)	0	18	7.81 (4.08)	0	18	10.7 (0.8)	^a^ *p* = .0002** ^b^ *p* = .3043, NS ^c^ *p =* .0001**
**Section II** ***Secretomotor and Sensory Integration (SS)***	7.37 (3.82)	0	18	5.66 (3.63)	1	19	4.5 (0.8)	^a^ *p* = .0352* ^b^ *p* = .0001** ^c^ *p* = .0625, NS
**Section III** ***Urinary and Gastrointestinal Systems (UG)***	5.19 (3.76)	0	15	4.55 (3.09)	0	12	3.8 (0.7)	^a^ *p* = .3945, NS ^b^ *p* = .0611, NS ^c^ *p* = .1434, NS
**Section IV** ***Circulation, Thermoregulation, and Sleep (CTS)***	4.27 (2.99)	0	17	3.94 (3.03)	0	14	3.1 (0.7)	^a^ *p* = .6132, NS ^b^ *p* = .0492* ^c^ *p* = .2676, NS

^a^ Group comparisons between mean PASS scores in probands with ASD (NPD‐ASD) as compared to mean PASS scores in probands with other forms of neurodevelopmental disorders (NPD‐OTHER). *p*‐Values reported adjusted via Bonferroni methods.

^b^ Group comparisons between mean PASS scores in probands with ASD (NPD‐ASD) as compared to individuals with ASD reported in Ming et al. (2011). *p*‐Values reported based on Wilcoxon signed test with continuity correction.

^c^ Group comparisons between mean PASS scores in probands with other forms of neurodevelopmental disorders (NPD‐OTHER) and individuals with ASD reported in Ming et al. (2011). *p*‐Values reported based on Wilcoxon signed test with continuity correction.

* *p* < .05;

** *p* < .001.

### 3.2 Individual differences in autonomic features associated with core clinical ASD features

**FIGURE 2:**
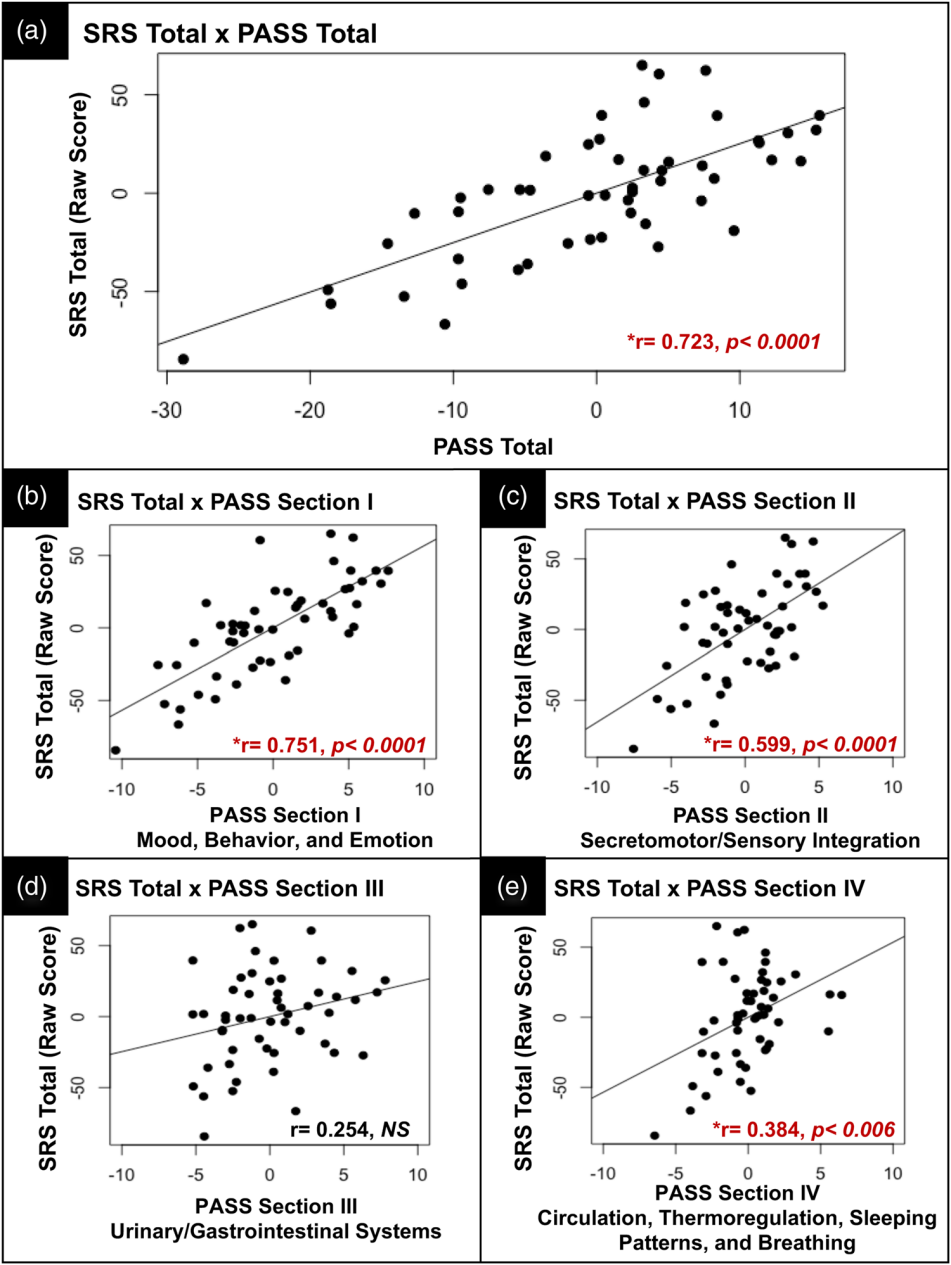
Results from Pearson, pairwise comparisons between SRS total (raw scores) and PASS total (A) and subscale scores (B–E)

Next, we assessed the relationship between autonomic features and quantitative traits of ASD across our NPD cohort by characterizing the relationship between the PASS and available SRS scores from *n* = 52 of 90 NPD (see Table 5) via partial correlation controlling for the combined effects of age and sex. PASS Total scores were significantly associated with SRS Total raw scores (*r* = 0.723, *p* < .0001) as well as all subscale scores (*p*’s < .0001) (see Figure 2). PASS (I) Mood, Behavior, and Emotion and (II) Secretomotor/Sensory Integration subscales were significantly related to Total and subscale SRS raw scores (*p*’s < .007). PASS (III) Urinary/Gastrointestinal Systems subscale scores were significantly related to SRS RRB and Social Awareness scores (*p*’s < .038), but no other SRS subscale scores. Finally, PASS (IV) Circulation, Thermoregulation, Sleeping Patterns, and Breathing subscale scores were significantly related to SRS Total raw scores (*r* = 0.384, *p* < .006) as well as subscale scores (*p*’s < .023) with the exception of Social Awareness and Social Motivation scores. See Table 5 for complete results from all correlation analyses between PASS and SRS raw scores. Finally, we also assessed the relationship between PASS and SRS scores separately within each of the NPD‐ASD and NPD‐OTHER subgroups. Complete results from this analysis can be found Tables S5 and S6.

**TABLE 5:** Partial correlation between SRS raw scores and PASS (age, sex) in NPD with genetic syndrome; corrected p‐values reported below

	PASS	PASS Section I		PASS Section II	PASS Section III		PASS Section IV
	Total	MBE		SS	UG		CTS
**SRS‐2 (Raw scores)**							
**Total Score**	**0.723, *p* < .0001** **	**0.751, *p* < .0001** **	**0.600, *p* < .0001** **		0.254, *p* = .076	**0.384, *p* = .006** *	
**SCI**	**0.702, *p* < .0001** **	**0.732, *p* < .0001** **	**0.586, *p* = .0001** **		0.230, *p* = .108	**0.388, *p* = .005** *	
**RBRI**	**0.692, *p* < .0001** **	**0.713, *p* < .0001** **	**0.562, *p* = .0002** **		**0.295, *p* = .038** *	**0.320, *p* = .023** *	
**Social awareness**	**0.569, *p* < .0001** **	**0.575, *p* = .0001** **	**0.499, *p* = .0002** **		**0.294, *p* = .038** *	0.165, *p* = .252	
**Social cognition**	**0.715, *p* < .0001** **	**0.765, *p* < .0001** **	**0.582, *p* = .0001** **		0.188, *p* = .191	**0.443, *p* = .001** **	
**Social communication**	**0.689, *p* < .0001** **	**0.660, *p* < .0001** **	**0.587, *p* = .0001** **		0.268, *p* = .059	**0.412, *p* = .003** *	
**Social motivation**	**0.478, *p <* .0001** **	**0.604, *p* < .0001** **	**0.374, *p* = .007** *		0.052, *p* = .717	0.249, *p* = .081	

Corrected *p*‐values reported below.

MBE, Mood, Behavior, and Emotion; SS, Secretomotor/Sensory Integration; UG, Urinary/Gastrointestinal Systems; CTS, Circulation, Thermoregulation, Sleeping Patterns, and Breathing.

* *p* < 0.05;

** *p* < 0.001.

## 4 DISCUSSION

The current investigation aimed to assess features of autonomic dysfunction in children with an NPD‐related genetic etiology. We find elevated symptoms of autonomic dysfunction in this cohort, including those with ASD, as compared to a reference sample of children with ASD of unknown etiology and healthy controls (Ming et al., 2011). Our reported results align with current research on symptoms indicative of autonomic dysfunction such as disrupted sleep patterns as well as gastrointestinal disturbances in NPD‐related genetic disorders, including 16p11.2 del and dup syndrome, Smith Magenis Syndrome, and 15q duplication syndrome, all of which were represented within our current cohort (Brunetti‐Pierri et al., 2008; Cerminara et al., 2021; Kamara et al., 2021; Leader et al., 2021; Shayota & Elsea, 2019). Previous work in ASD either has relied only on clinical diagnoses for inclusion into a study (ignoring genetic etiology) or focused on one‐specific genetic etiology and associated NPD diagnoses (Angkustsiri et al., 2014; Fine et al., 2005; Kumar et al., 2008; Martin et al., 2020; Moreno‐De‐Luca et al., 2013; Moreno‐De‐Luca et al., 2015). Because individual NPD‐related genetic disorders are individually rare, amassing a large cohort with the same genetic etiology is challenging. Scalable methods such as meta‐analyses and curation of existing phenotypic data in larger samples will be necessary to move the field forward toward identifying behavioral domains that may be specifically impacted based on distinct chromosomal anomalies.

While we have ruled out the possible effect of age as a reason for the significant differences in PASS scores within our NPD cohort and a younger reference sample of children with ASD (Ming et al., 2011), there are other possible reasons for our reported results. In addition to being a larger cohort that includes a wider age range, our sample includes a broad spectrum of clinical diagnoses and genetic etiologies. It is also important to highlight the broad scope of ASD traits, assessed via the SRS, in our NPD cohort and across those with and without ASD. SRS raw scores were used in analyses as a measure of ASD traits in order to maximize phenotypic variability observed across our sample. Less than 25% of our NPD group presented with symptoms in the severe range according to SRS T‐scores (see Table 2) and several individuals had co‐occurring diagnoses of mild‐to‐severe intellectual disability (see Table S1). While phenotypic information was not reported as a part of Ming et al. (2011), the current investigation includes individuals from across the diagnostic spectrum, especially those with a more severe clinical presentation (i.e., those that would be clinically described as lower functioning). The wide range of phenotypic traits, ranging from mild to severe, was also critical in analysis regarding the association between SRS and PASS scores. Thus, the heterogeneous and variable phenotypes, including the various genetic etiologies, present within our reported cohort are a major distinction between the current investigation and previous studies using the PASS (Ming et al., 2011). However, it is important to note that comorbidities and genetic diagnoses (with the exception of those with a targeted genetic etiology associated with familial dysautonomia) were not reported in previous published work (i.e., Ming et al., 2011).

Recent research has emphasized the important role of genetics in understanding the variable development and expression of core clinical features of ASD and other NPD. For example, the phenotypic outcomes of high‐penetrance genetic disorders (such as CNVs or single gene disorders) that cause NPD are influenced by other genetic background variance (Finucane et al., 2016; Moreno‐De‐Luca et al., 2015). Recent work on transdiagnostic traits, such as "externalizing" behaviors (Linnér et al., 2019) have proven to be successful at capturing larger amounts of genetic variance than models that strictly rely on clinical diagnostic categories. Thus, there is growing consensus that clinical nosology may need to be set aside in favor of transdiagnostic traits (Dalgleish et al., 2020). Despite this growing realization, research on psychiatric and neurodevelopmental traits has continued to be siloed based on clinically defined diagnostic boundaries. Other work has taken a genetics‐first approach to understanding gene‐behavior relationships (Moreno‐De‐Luca et al., 2015), but these two approaches (diagnosis/phenotype approach vs. genotype‐first approach) can be challenging to marry in real‐world clinical settings. Here, we demonstrate a strategy for sampling and analysis of a clinically heterogeneous population that, if applied to future studies, may help the field overcome limitations of small sample sizes when focusing on homogeneous and rare disease groups. The concurrent use of clinical data, genetic diagnoses, and transdiagnostic trait assessment has the potential to inform correlations between pathogenic variants associated with NPDs and patient phenotypes (Kothari et al., 2018).

The work presented here uses a broad sampling approach that aimed to address whether there is a relationship between ANS dysfunction and ASD traits, while also using a real‐world heterogeneous, genetically characterized cohort. From our perspective, individual differences in autonomic features may represent a measurable, transdiagnostic trait domain that is more closely linked to underlying mechanistic drivers of atypical neurodevelopmental features. Outside of experimental studies of functional autonomic processes (Condy et al., 2017; Kushki et al., 2013; Kushki et al., 2014; Patriquin et al., 2013), features of ANS dysfunction have not been quantitatively assessed across ASD and other NPDs. To measure individual differences in autonomic features, we make use of a parent‐report measure, the PASS. We report a linear relationship between features of autonomic dysfunction (PASS Total and subscale scores) and ASD traits across probands with an identified NPD‐related genetic etiology. In addition to elevated autonomic symptoms across PASS subdomains, individual differences in autonomic features were significantly associated with clinically significant ASD traits. We wish to acknowledge that characterizing the causal relationship between psychosocial impairments and core clinical features of ASD and atypical autonomic processes is beyond the scope of the current study. However, given previous research on autonomic dysfunction in ASD and other studies linking specific autonomic processes such as HRV with psychosocial impairment and emotion regulation (Appelhans & Luecken, 2006; Bunford et al., 2017; Patriquin et al., 2013; Patriquin et al., 2019; Quintana et al., 2012; Thapa et al., 2021), this and the current results provide strong evidence for future work directly assessing the causality of autonomic function as a biological driver for neurodevelopmental traits.

Although this work is the first to capture the PASS scales in patients with a range of NPD, including ASD as well as other neurodevelopmental disorders, other measures have been used to assess symptoms that have been linked with autonomic processes (Bernstein et al., 1997; Woodard et al., 2012). Previous work that has used functional autonomic indices within an experimental context is able to determine whether there is increased or decreased ANS activity in ASD. However, existing results are discordant (Kushki et al., 2014; Wang et al., 2016), with some studies identifying increased sympathetic versus parasympathetic activity in ASD and related NPDs and others finding the opposite relationship (Anderson & Colombo, 2009; Billeci et al., 2018; Condy et al., 2017; Klusek et al., 2015; Neuhaus et al., 2014; Patriquin et al., 2013; Patriquin et al., 2019; Wang et al., 2016). Previous research has also investigated distinct autonomic symptoms (i.e., gastrointestinal problems and sleep disturbances assessed via parent‐report), in conjunction with core features of ASD, as a possible way to stratify clinically meaningful subgroups (Unwin et al., 2013). However, in the current investigation and based on the high inter‐correlations between PASS subscales (see Table S4), it is unclear whether distinct autonomic phenotypes are presented within our reported clinical cohort or if ASD symptoms are more broadly associated with autonomic dysregulation. Furthermore, the PASS does not allow us to specify increased or decreased activity across organ systems or within specific autonomic processes (i.e., sympathetic vs. parasympathetic). Thus, although this work represents the potential for expanded use of the PASS as a quantitative index of autonomic features, additional research in larger samples that links autonomic symptoms, ASD traits, and functional metrics of ANS dysfunction will be an important next step to (i) characterize autonomic function as a biological driver for atypical neurodevelopment and (ii) identify distinct autonomic processes that may differentiate clinical subtypes in neurodevelopmental populations including ASD.

Our sample was characterized by a combination of CNVs and single gene disorders, with a few CNVs with uncertain pathogenicity. Advances in clinical genomic testing have identified numerous pathogenic variants causative for ASD and other complex neurodevelopmental and psychiatric phenotypes; however, characterization of variants of uncertain significance and the discovery of genes and mechanistic pathways connected to neural, cognitive, and biological processes are ongoing (De Rubeis et al., 2014; Iossifov et al., 2014). Genetic variants associated with ASD also confer risk for other neuropsychiatric conditions, medical problems, and other diseases (i.e., ataxias, altered growth patterns, hearing and/or visual impairments) and atypical function in various organ systems regulated by the ANS (Cappuccio et al., 2016; Daghsni et al., 2018; Lecavalier et al., 2019; Robinson et al., 2016; Talkowski et al., 2011; Zhang et al., 2021). The continued study and characterization of symptom dimensions that may be transdiagnostic is an important next step in understanding the underlying biology of known and emerging genetic disorders with complex neurodevelopmental phenotypes (Savatt & Myers, 2021; Srivastava et al., 2019).

This study has several limitations. The current sample included a broad age range that extended beyond previously published results that used the PASS in younger children (Ming et al., 2011). We explored potential age‐related differences in PASS scores across our sample and were unable to identify a main effect of age. Thus, our results represent an expanded approach and increase the generalizability of our findings. While this may be viewed as a relative strength, it is important to acknowledge that the original published use of the PASS was in a much narrower and younger age group (1). In future iterations or as a part of larger studies, the item structure and wording within specific PASS questions should be considered and adapted as needed for a wider age range and to be more developmentally appropriate (i.e., “Does he or she line up toys or other objects…?; Does your child ever tell you he/she feels ‘‘big in the tummy” or bloated?). We also wish to acknowledge that our study design and the included sample were not directly based on a priori power analyses and/or sample size calculations. The current work was supported by an administrative supplement as a part of ongoing NIH R01 (see funding information listed in Acknowledgments) focused on characterizing the impact of CNVs and other genetic variants on neurodevelopmental traits). As stated above, measures of interest (i.e., the PASS) were included in an online phenotyping battery that was administered to already consented parents/caregivers. Thus, we acknowledge that our reported results are based on a convenience sample; however, our included sample sizes are larger than previously published reference samples highlighted in the current investigation (Ming et al., 2011). Additionally, a primary objective of the current study was to quantitatively assess individual differences in autonomic symptoms across multiple organ systems in a heterogeneous cohort, a method and research objective that is in contrast to other studies that have investigated isolated processes of ANS function (i.e., HRV, tonic pupil size) and in smaller samples (Anderson & Colombo, 2009; Condy et al., 2017; Kushki et al., 2014; Ming et al., 2005). Despite this, it will be critical in future studies to perform appropriate sample size calculations and power analyses in order to accurately address research objective and maximize effect sizes. Also due to the nature of the ongoing research funding and the corresponding project, we relied on a combination of parent‐report medical history and clinical data from individuals’ EHR documentation from expert neurodevelopmental diagnosticians at our clinic. This strategy allowed us to eliminate time and participant burden typically required for in‐person testing, but limited comprehensive assessment of other traits, such as cognitive ability. Although this is certainly a limitation, this strategy does allow for inclusion of a more diverse neurodevelopmental population, including some children that may be unable to complete in‐person research testing due to more profound impairment. In contrast to studies that tend to rely on in‐person human subjects testing methods, the strategy used here is amenable to cost‐effective scaling and can be more inclusive to those with more severe cognitive and behavioral impairments that tend to be excluded from traditional in‐person research testing. Furthermore, this approach allows for the capture of meaningful variability in autonomic processes across the full spectrum of NPD. Finally, we wish to acknowledge that the current investigation did not include specific subgroup analyses of genetic variants included in our cohort that have previously been reported to present with atypical autonomic features (Brunetti‐Pierri et al., 2008; Cerminara et al., 2021; Kamara et al., 2021; Leader et al., 2021; Shayota & Elsea, 2019) (e.g., comparing PASS scores and/or assessing PASSxSRS correlations between targeted genetic variants). Smaller samples sizes, even in the more highly represented genetic variants within our cohort (i.e., 16p11.2 del syndrome, *n* = 14), left us underpowered to draw meaningful group comparisons and identify significant results. While collapsing deletions and duplications within some of our variant subgroups would increase our *n*’s and power in identifying significant group differences, these alterations are complex with deletions and duplications often leading to highly variable phenotypes (Chawner et al., 2021; Kates et al., 2007; Wenger et al., 2016) and clinical features even within the same variant. Thus, while we see this as an exciting future direction for this work and a critical next step in characterizing the genetic contribution to autonomic dysfunction in neurodevelopmental populations, these analyses were beyond the scope of the current study.

This study quantifies symptoms of autonomic dysfunction in a heterogeneous, neurodevelopmental pediatric cohort characterized based on both clinical diagnoses as well as clinical genetic etiology. Thus, our sampling methods and study findings move toward the characterization of autonomic dysfunction as a transdiagnostic trait within atypical neurodevelopment, including ASD, rather than within a specific diagnostic group (Anderson & Colombo, 2009; Billeci et al., 2018; Condy et al., 2017; Ming et al., 2005; Pace et al., 2016). Results from the current research lay the groundwork for future studies aimed at characterizing individual differences in atypical autonomic processes as important transdiagnostic traits that are associated with clinically significant neurodevelopmental symptoms. Further characterization of the link between ANS functional, neurodevelopmental, and genetic differences is an important next step toward understanding the biological pathways underlying NPD and may lead to the identification of clinical subtypes and objective biomarkers, based on symptomology and genetic etiology that are amenable to novel treatment targets.

## CONFLICT OF INTEREST

The authors declare that they have no competing interests.

## AUTHOR CONTRIBUTIONS

DiCriscio and Troiani had full access to all of the data in the study and take responsibility for the integrity of the data and the accuracy of the data analysis. Concept and design: DiCriscio and Troiani. Acquisition, analysis, or interpretation of data: DiCriscio, Smith, Beiler, Walsh, Holdren, Wain, and Troiani. Drafting of the manuscript: DiCriscio, Smith, and Troiani. Critical revision of the manuscript for important intellectual content: DiCriscio, Wain, and Troiani. Statistical analysis: DiCriscio. Obtained funding: DiCriscio and Troiani. Administrative, technical, or material support: DiCriscio, Smith, Beiler, Walsh, Holdren, Wain, and Troiani.

### PEER REVIEW

The peer review history for this article is available at https://publons.com/publon/10.1002/brb3.2813

## Supporting information

Supplemental Table 1: Clinical diagnosis in NPD‐ASD and NPD‐OTHER subgroups
Supplemental Table 2: Demographics and sample characteristics
Supplemental Table 3: Age based analysis of PASS
Supplemental Table 4: Inter‐correlations between PASS sections
Supplemental Table 5: PASS and SRS correlations, NPD‐ASD
Supplemental Table 6: PASS and SRS correlations, NPD‐OTHER

## Data Availability

Data are available on request due to privacy/ethical restrictions
