# Supplementary material for: Higher scores on autonomic symptom scales in pediatric patients with neurodevelopmental disorders of known genetic etiology
Source: Brain Behav. 2022 Nov 24;12(12):e2813. doi: 10.1002/brb3.2813 (PMC9759134; doi:10.1002/brb3.2813)
Supplement: Supplementary file 1 — Supplemental Table 1: Clinical diagnosis in NPD‐ASD and NPD‐OTHER subgroups Supplemental Table 2: Demographics and sample characteristics Supplemental Table 3: Age based analysis of PASS Supplemental Table 4: Inter‐correlations between PASS sections Supplemental Table 5: PASS and SRS correlations, NPD‐ASD Supplemental Table 6: PASS and SRS correlations, NPD‐OTHER [file BRB3-12-e2813-s001.docx]

**SUPPLEMENTAL MATERIAL**

**TITLE**: Higher scores on autonomic symptoms scales in pediatric patients with neurodevelopmental disorders of known genetic etiology

Table of Contents

Supplemental Table 1: Clinical diagnosis in NPD-ASD and NPD-OTHER subgroups…………………....1

Supplemental Table 2: *Available scores on Child Behavior Checklist (CBCL)..……………………………...*2

Supplemental Table 3: *Inter-correlations between PASS sections………………………………………………*3

Supplemental Table 4: *Age based analysis of PASS……………………………………………………………….*4

Results from supplemental analysis*…………………………………………………………………………*4

Table ..*…………………………………………………………………………………………………………..*5

Supplemental Table 5: *PASS and SRS correlations, NPD-ASD………………………………………………....*6

Supplemental Table 6: *PASS and SRS correlations, NPD-OTHER……………………………………………..*7

**Supplemental Table 1.** Clinical diagnoses within the NPD-ASD (n=37 of 90) and NPD-OTHER (n=53 of 90) subgroups.

| **Clinical Diagnosis** | **NPD-ASD n=37 of 90** | **NPD-OTHER**  **n= 53 of 90** |
| --- | --- | --- |
| **Autism Spectrum Disorder (ASD)* | 37 | 0 |
| Mixed receptive-expressive language disorder | 14 | 18 |
| Attention Deficit Hyperactivity Disorder | 11 | 18 |
| Anxiety disorder | 11 | 13 |
| Unspecified Intellectual Disability (ID) | 7 | 12 |
| Developmental delay | 5 | 10 |
| Speech sound disorder | 6 | 9 |
| Coordination disorder | 9 | 8 |
| Global Developmental Delay | 4 | 6 |
| Learning disorder | 1 | 6 |
| Seizure disorder | 4 | 4 |
| Mild ID | 3 | 4 |
| Cognitive impairment | 3 | 4 |
| Mood disorder | 1 | 4 |
| Social communication disorder | 5 | 3 |
| Minimally verbal | 1 | 3 |
| Epilepsy | 0 | 3 |
| Borderline ID | 0 | 3 |
| Phonological disorder | 0 | 3 |
| Sensory integration disorder and/or processing disorder | 0 | 2 |
| Speech delay | 2 | 2 |
| Childhood apraxia of speech | 1 | 2 |
| Self-injurious behavior | 1 | 2 |
| Moderate ID | 2 | 1 |
| Nonverbal | 1 | 1 |
| Obsessive Compulsive Disorder | 1 | 1 |
| Oppositional defiant disorder | 1 | 1 |
| Stuttering | 1 | 1 |
| Disruptive behavior disorder | 1 | 1 |
| Bipolar disorder | 0 | 1 |
| Pervasive Developmental Disorder-NOS (defined by DSM IV) | 0 | 1 |
| Developmental language disorder | 0 | 1 |
| Cerebral palsy | 0 | 1 |
| Psychosis Not Otherwise Specified (NOS) | 0 | 1 |
| Mild-Moderate ID | 0 | 1 |
| Expressive language disorder | 0 | 1 |
| Communication disorder NOS | 0 | 1 |
| Articulation disorder | 2 | 0 |
| Other symbolic dysfunction | 2 | 0 |
| Stereotypy | 2 | 0 |
| Severe ID | 2 | 0 |
| **Supplemental Table 1** *continued* |  |  |
| **Clinical Diagnosis** | **NPD-ASD n=37 of 90** | **NPD-OTHER**  **n= 53 of 90** |
| Semantic-pragmatic disorder | 1 | 0 |
| Conductive hearing loss | 1 | 0 |
| Hyperkinesis | 1 | 0 |

**Supplemental Table 2.** Available CBCL scores in n= 43 of 90 NPD (27 males)

|  | $\bar{\boldsymbol{x}}\boldsymbol{(\sigma)}$ | Min | Max |
| --- | --- | --- | --- |
| **CBCL School Age t-scores (n=26; Male: 17)** |  |  |  |
| Total | 65.38 (8.72) | 48 | 83 |
| Anxious/Depressed | 56.96 (9.15) | 50 | 79 |
| Withdrawn/Depressed | 63.85 (8.97) | 50 | 78 |
| Somatic Complaints | 60.65 (8.85) | 50 | 85 |
| Rule Breaking | 57.73 (6.58) | 50 | 81 |
| Aggressive Behavior | 61.65 (9.17) | 50 | 89 |
| Social Problems | 65.85 (8.06) | 51 | 88 |
| Thought Problems | 66.27 (9.97) | 50 | 88 |
| Attention Problems | 69.60 (10.43) | 51 | 87 |
| **CBCL Preschool Age t-scores (n=17; Male:10)** |  |  |  |
| Total | 63.06 (16.09) | 31 | 88 |
| Emotionally Reactive | 63.24 (12.84) | 50 | 93 |
| Anxious/Depressed | 57.24 (9.86) | 50 | 83 |
| Somatic Complaints | 56.47 (6.99) | 50 | 72 |
| Withdrawn/Depressed | 66.41 (10.86) | 50 | 85 |
| Attention Problems | 66.65 (9.51) | 50 | 80 |
| Aggressive Behavior | 63.82 (12.59) | 50 | 88 |
| Sleep Problems | 61.24 (10.89) | 50 | 88 |

**Supplemental Table 3.** Partial inter-correlations (age and sex) between PASS subsection and total scores in N=90 NPD, controlling for age and sex. All *p*-values are adjusted for multiple comparisons via Bonferroni methods.

|  | **PASS Total** | **PASS Section I MBE** | **PASS Section II SS** | **PASS Section III UG** | **PASS Section IV CTS** |
| --- | --- | --- | --- | --- | --- |
| **PASS Total** | 1.00, - |  |  |  |  |
| **PASS Section I MBE** | **0.818, *p*<0.0001*** | 1.00, - |  |  |  |
| **PASS Section II SS** | **0.869, *p*<0.0001*** | **0.681, *p*<0.0001*** | 1.00, - |  |  |
| **PASS Section III UG** | **0.621, *p*<0.0001*** | **0.235, *p*=0.0278*** | **0.394, *p*=0.0002*** | 1.00, - |  |
| **PASS Section IV CTS** | **0.719, *p*<0.0001*** | **0.434, *p*<0.0001*** | **0.521, *p*<0.0001*** | **0.345, *p*=0.0011*** | 1.00, - |

**Supplemental Table 4.** It is important to note that Ming et al. (68) reported PASS scores in children ages 2 thru 5 years of age. In the current study, we have extended this age range up to 18 years of age. Thus, we explored potential age effects on PASS scores in the current reported sample of pediatric probands. Our cohort of pediatric NPD probands was divided into subgroups according to age: 2-5 years; 6-10 years; and 11-18 years. An analysis of variance (ANOVA) was used to determine if PASS scores differed by age in our reported sample. Results did not indicate a main effect of age on PASS Total (F(2,87)=0.470, *p*=0.627, NS) or PASS subscale scores (*p*’s>0.391, NS). We also assessed relationships between our quantitative measures and age; however, age was not found to be associated with our parent-report measures (i.e., PASS, SRS) (*p*’s>0.097, NS).

Thus, while our current approach extended the use of the PASS across a broader pediatric age range, autonomic symptoms did not differ across age groups. Supplemental Table 2 (see below) includes group averages for PASS Total and subscale scores across age groups described above.

**Supplemental Table 4 (continued).** PASS scores in probands (N=90 NPD) according to age: 2-5 years old (yo); 6-10 yo; and 10-18 yo. Group average PASS scores reported in n=18 with ASD in Ming et al. (2011) (68) also included (last column).

|  | **N=90 NPD** | | | | | | | | | | |  | **Ming et al. (2011)** |
| --- | --- | --- | --- | --- | --- | --- | --- | --- | --- | --- | --- | --- | --- |
|  | **2-5 yo**  **(n=18 of 90)** | | |  | **6-10 yo**  **(n=33 of 90)** | | |  | **10-18 yo**  **(n=39 of 90)** | | |  | **2-5 yo**  **(n=18 with ASD)** |
|  | $\bar{\boldsymbol{x}}\boldsymbol{(\sigma)}$ | **Min** | **Max** |  | $\bar{\boldsymbol{x}}\boldsymbol{(\sigma)}$ | **Min** | **Max** |  | $\bar{\boldsymbol{x}}\boldsymbol{(\sigma)}$ | **Min** | **Max** |  | $\bar{\boldsymbol{x}}\boldsymbol{(\sigma)}$ |
| **PASS Total** | 22.39 (13.31) | 9 | 68 |  | 24.21 (10.14) | 5 | 45 |  | 25.74 (10.99) | 3 | 43 |  | 20.6 (2.7) |
| **Section I**  ***Mood, Behavior, & Emotion (MBE)*** | 7.4 (4.52) | 0 | 18 |  | 9.36 (4.20) | 1 | 17 |  | 9.69 (4.90) | 0 | 18 |  | 10.7 (0.8) |
| **Section II**  ***Secretomotor & Sensory Integration (SS)*** | 5.2 (4.07) | 0 | 18 |  | 6.46 (3.91) | 1 | 19 |  | 6.69 (3.53) | 0 | 12 |  | 4.5 (0.8) |
| **Section III**  ***Urinary & Gastrointestinal Systems (UG)*** | 4.90 (3.34) | 1 | 15 |  | 4.55 (3.26) | 0 | 11 |  | 4.80 (3.43) | 0 | 13 |  | 3.8 (0.7) |
| **Section IV**  ***Circulation, Thermoregulation, & Sleep (CTS)*** | 3.20 (3.50) | 0 | 17 |  | 3.85 (2.55) | 0 | 11 |  | 4.56 (2.95) | 0 | 14 |  | 3.1 (0.7) |

**Supplemental Table 5.** Partial correlations between SRS raw scores and PASS in N=23 NPD-ASD, controlling for age and sex. All *p*-values are adjusted for multiple comparisons.

|  | **PASS**  **Total** | **PASS Section I**  **MBE** | | | **PASS Section II**  **SS** | **PASS Section III**  **UG** | | | **PASS Section IV**  **CTS** |
| --- | --- | --- | --- | --- | --- | --- | --- | --- | --- |
| **SRS-2 (Raw scores)** |  | | | | | | | | |
| **Total Score** | **0.583, *p*=0.0203*** | | **0.598, *p*=0.0182*** | **0.623, *p*=0.0149*** | | | 0.193, *p*=0.5610 | -0.012, *p*=0.9776 | |
| **SCI** | **0.580, *p*=0.0203*** | | **0.615, *p*=0.0152*** | **0.623, *p*=0.0149*** | | | 0.177, *p*=0.5969 | -0.025, *p*=0.9776 | |
| **RBRI** | 0.484, *p*=0.0541 | | 0.433, *p*=0.0969 | **0.507, *p*=0.0444*** | | | 0.213, *p*=0.5274 | 0.031, *p*=0.9776 | |
| **Social Awareness** | 0.487, *p*=0.0541 | | 0.320, *p*=0.2757 | **0.559, *p*=0.0227*** | | | 0.261, *p*=0.4039 | 0.086, *p*=0.8572 | |
| **Social Cognition** | **0.642, *p*=0.0149*** | | **0.699, *p*=0.0089*** | **0.692, *p*=0.0089*** | | | 0.165, *p*=0.6160 | -0.007, *p*=0.9776 | |
| **Social Communication** | **0.574, *p*=0.0208*** | | **0.540, *p*=0.0287*** | **0.643, *p*=0.0149*** | | | 0.210, *p*=0.5274 | -0.009, *p*=0.9776 | |
| **Social Motivation** | 0.344, *p*=0.2337 | | **0.560, *p*=0.0227*** | 0.305, *p*=0.2990 | | | 0.012, *p*=0.9776 | -0.110, *p*=0.7931 | |

**Supplemental Table 6.** Partial correlations between SRS raw scores and PASS in N=29 NPD-OTHER, controlling for age and sex. All *p*-values are adjusted for multiple comparisons.

|  | **PASS**  **Total** | **PASS Section I**  **MBE** | | | **PASS Section II**  **SS** | **PASS Section III**  **UG** | | | **PASS Section IV**  **CTS** |
| --- | --- | --- | --- | --- | --- | --- | --- | --- | --- |
| **SRS-2 (Raw scores)** |  | | | | | | | | |
| **Total Score** | **0.738, *p*=0.0002*** | | **0.704, *p*=0.0003*** | **0.558, *p*=0.0049*** | | | 0.225, *p*=0.2847 | **0.667, *p*=0.0005*** | |
| **SCI** | **0.690, *p*=0.0004*** | | **0.663, *p*=0.0005**** | **0.515, *p*=0.0108*** | | | 0.188, *p*=0.3695 | **0.649, *p*=0.0007*** | |
| **RBRI** | **0.767, *p*=0.0001*** | | **0.714, *p*=0.0003*** | **0.603, *p*=0.0021*** | | | 0.316, *p*=0.1356 | **0.602, *p*=0.0021*** | |
| **Social Awareness** | **0.494, *p*=0.0147*** | | **0.491, *p*=0.0147*** | **0.406, *p*=0.0480*** | | | 0.267, *p*=0.2158 | 0.251, *p*=0.2410 | |
| **Social Cognition** | **0.688, *p*=0.0004*** | | **0.722, *p*=0.0002*** | **0.513, *p*=0.0108*** | | | 0.143, *p*=0.4923 | **0.611, *p*=0.0019*** | |
| **Social Communication** | **0.671, *p*=0.0005*** | | **0.591, *p*=0.0026*** | **0.479, *p*=0.0174*** | | | 0.229, *p*=0.2829 | **0.678, *p*=0.0004*** | |
| **Social Motivation** | **0.455, *p*=0.0243*** | | **0.454, *p*=0.0243*** | 0.363, *p*=0.0818 | | | -0.031, *p*=0.8796 | **0.558, *p*=0.0049*** | |
